# ABO Blood Groups Influence Macrophage-mediated Phagocytosis of *Plasmodium falciparum*-infected Erythrocytes

**DOI:** 10.1371/journal.ppat.1002942

**Published:** 2012-10-11

**Authors:** Kayla T. Wolofsky, Kodjo Ayi, Donald R. Branch, Annika K. Hult, Martin L. Olsson, W. Conrad Liles, Christine M. Cserti-Gazdewich, Kevin C. Kain

**Affiliations:** 1 Sandra Rotman Centre for Global Health, SA Rotman Laboratories, Toronto General Hospital-University Health Network, University of Toronto, Toronto, Ontario, Canada; 2 Institute of Medical Science, University of Toronto, Toronto, Ontario, Canada; 3 Research and Development, Canadian Blood Services, Toronto, Ontario, Canada; 4 Division of Hematology and Transfusion Medicine, Department of Laboratory Medicine, Lund University, Lund, Sweden; 5 Department of Medicine, University of Washington, Seattle, Washington, United States of America; 6 Tropical Disease Unit, Division of Infectious Diseases, Department of Medicine, Toronto General Hospital-University Health Network, University of Toronto, Toronto, Ontario, Canada; 7 Blood Transfusion Laboratory, Toronto General Hospital-UHN, Toronto, Ontario, Canada; National Institute for Medical Research, United Kingdom

## Abstract

Erythrocyte polymorphisms associated with a survival advantage to *Plasmodium falciparum* infection have undergone positive selection. There is a predominance of blood group O in malaria-endemic regions, and several lines of evidence suggest that ABO blood groups may influence the outcome of *P. falciparum* infection. Based on the hypothesis that enhanced innate clearance of infected polymorphic erythrocytes is associated with protection from severe malaria, we investigated whether *P. falciparum*-infected O erythrocytes are more efficiently cleared by macrophages than infected A and B erythrocytes. We show that human macrophages *in vitro* and mouse monocytes *in vivo* phagocytose *P. falciparum*-infected O erythrocytes more avidly than infected A and B erythrocytes and that uptake is associated with increased hemichrome deposition and high molecular weight band 3 aggregates in infected O erythrocytes. Using infected A_1_, A_2_, and O erythrocytes, we demonstrate an inverse association of phagocytic capacity with the amount of A antigen on the surface of infected erythrocytes. Finally, we report that enzymatic conversion of B erythrocytes to type as O before infection significantly enhances their uptake by macrophages to observed level comparable to that with infected O wild-type erythrocytes. These data provide the first evidence that ABO blood group antigens influence macrophage clearance of *P. falciparum*-infected erythrocytes and suggest an additional mechanism by which blood group O may confer resistance to severe malaria.

## Author Summary

*Plasmodium falciparum* malaria is considered to be one of the strongest forces for evolutionary selection pressure on the human genome. Different red blood cell variants associated with a survival advantage to *P. falciparum* infection have undergone positive selection. Blood group O is found more frequently in malaria-endemic regions and has been associated with protection against severe malaria and death. However the biological basis of protection remains unclear. In this study, we investigated innate immune clearance of *P. falciparum*-infected erythrocytes by macrophages as a possible mode of protection. We show that macrophages clear *P. falciparum*-infected O erythrocytes more avidly than infected A and B erythrocytes. We also report that enzymatic conversion of infected blood group B red cells to type as “O” like erythrocytes significantly enhances their uptake by macrophages to a level comparable to that observed with infected O wild type erythrocytes. These data provide the first evidence that clearance of *P. falciparum*-infected erythrocytes is influenced by human ABO blood groups and suggest a new mechanism by which blood group O may contribute to protection against severe malaria.

## Introduction

*Plasmodium falciparum* malaria is responsible for an estimated 1.24 million deaths annually, with the majority of deaths occurring in individuals before reproductive age [1]. *P. falciparum* malaria predated the development of modern *Homo sapiens* and has co-evolved with human populations [2], [3]. It is considered to be one of the strongest forces for evolutionary selection of the human genome [2], [3]. In populations where *P. falciparum* infection is highly prevalent, common erythrocyte polymorphisms, such as deficiencies in globin synthesis, membrane proteins and erythrocyte enzymes, are associated with protection against severe and fatal disease [4]–[6].

Recent evidence suggests that the ABO blood group system has also been subject to malaria-related selection pressure [2], [3]. The ABO phenotype is determined by a polymorphic gene that encodes an enzyme, ABO glycosyltransferase that conjugates A- or B-specific sugar residues onto the precursor molecule known as the H antigen. If functionally active ABO glycosyltransferase is inherited via the co-dominant *A* or *B* alleles, transfer of either α-1,3-linked *N-*acetylgalactosamine or α-1,3-linked galactose produces A and B antigens, respectively, resulting in the non-O blood groups (A, B and AB). Molecular evidence indicates that the predominant *O* allele arose as the result of a loss-of-function mutation at nucleotide position 261 [7], [8]. Consequently, in O erythrocytes, the H antigen is left unaltered and ends with an α-1,2-linked fucose moiety that lacks the terminal α-1,3-linked monosaccharides [9].

Host pathogen interactions have been proposed as an important evolutionary force shaping the global distribution of ABO blood groups [10]. There is strong epidemiological evidence that the ABO phenotype may modulate disease severity and outcome of *P. falciparum* malaria, with blood groups A and B associated with increased disease severity compared to blood group O [11]–[15]. This association is consistent with the higher prevalence of group O observed in malaria-endemic sub-Saharan Africa compared to many parts of the world where malaria is not endemic, suggesting that blood group O may be a selected, protective adaptation against severe and fatal infection [2], [16], [17].

While several studies have reported that individuals with blood groups A and B are more likely to develop severe malaria, the mechanisms underlying the putative protection afforded by blood group O remain unclear [11], [12], [15]. Proposed mechanisms of protection parallel those implicated in other erythrocyte polymorphisms and include decreased erythrocyte invasion and reduced erythrocyte rosetting [12], [18]. Several studies have examined the association of ABO blood groups with rosetting and have reported that infected O erythrocytes exhibit fewer or smaller rosettes *in vitro* [12], [19]–[23] and *in vivo* [24] than infected A and B erythrocytes. Decreased rosetting may reduce microvascular obstruction that is believed to contribute to the pathogenesis of severe disease [12], [13], [16]. However, alternative mechanisms may also exist that contribute to the protective effect to *P. falciparum* malaria observed in individuals with blood group O.

*P. falciparum* infection and intracellular growth induce profound changes to the erythrocyte membrane resembling red cell senescence [25]. These changes, including hemichrome formation and band 3 aggregation resulting in erythrophagocytosis, may be accelerated in the presence of underlying erythrocyte disorders [26], [27]. Based on these observations, we hypothesized that enhanced senescence and phagocytosis of infected O erythrocytes, resulting in improved innate clearance and lower parasite densities, may provide an alternative explanation for protection observed in blood group O individuals. Studies of other erythrocyte polymorphisms associated with malaria-endemic areas, including sickle cell trait, beta-thalassemia, G6PD trait, and pyruvate kinase deficiency [26]–[28] that have reported increased phagocytosis of *P. falciparum*-infected variant erythrocytes, are also consistent with this hypothesis. Here, we show that *P. falciparum* parasites invade and mature similarly in group A, B and O erythrocytes. However, compared to *P. falciparum-*infected A and B erythrocytes, infected O erythrocytes display enhanced hemichrome deposition, band 3 aggregation and increased macrophage phagocytosis *in vitro* and *in vivo*. These data suggest that enhanced clearance of infected O erythrocytes may represent a novel mechanism by which blood group O contributes to protection against severe malaria.

## Results

### *P. falciparum* parasite invasion and maturation are similar in group A, B and O erythrocytes

**Figure 1:**
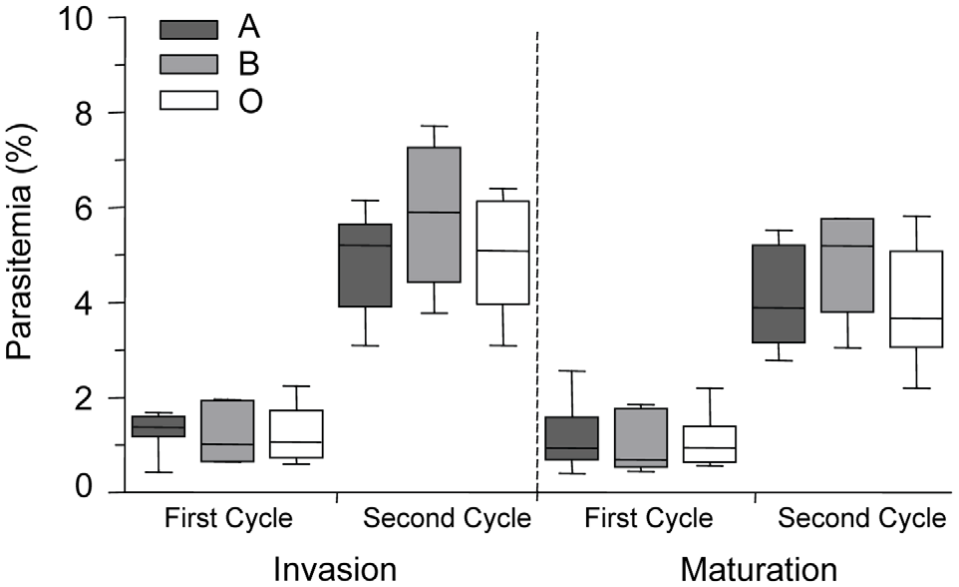
*P. falciparum* parasite invasion of A, B and O erythrocytes. Levels of parasite invasion and maturation during two cycles of growth of *P. falciparum* clone ITG in A, B and O erythrocytes. Data are presented as the combined results of 4 independent experiments using erythrocytes from healthy human volunteers with blood groups A (n = 7 donors), B (n = 4 donors) and O (n = 6 donors). Data are shown as box-and-whiskers plots, representing interquartile and complete ranges, with the horizontal line in each box indicating the median. Statistical significance was determined by a one way ANOVA. Invasion is defined as the percentage of ring parasitemia, as measured 24 hours and 72 hours after inoculation. Maturation is defined as the percentage of trophozoite parasitemia, as measured 48 hours and 96 hours after inoculation. There were no significant differences observed in the invasion and maturation of *P. falciparum* parasites in A, B or O erythrocytes.

To determine if ABO polymorphism influences malaria parasite invasion and maturation, we examined *P. falciparum* (ITG clone) parasite invasion and development in A, B, and O erythrocytes *in vitro*. No statistically significant differences in parasite invasion of group A, B and O erythrocytes were observed during two growth cycles (Figure 1). In addition, there were no significant differences noted in intracellular maturation (from ring stage to trophozoite stage parasites) within A, B and O erythrocytes (Figure 1). Similar results were obtained using two other clones (3D7, E8B) of *P. falciparum* malaria (data not shown).

### Infected O erythrocytes are more avidly phagocytosed than infected A or B erythrocytes

**Figure 2:**
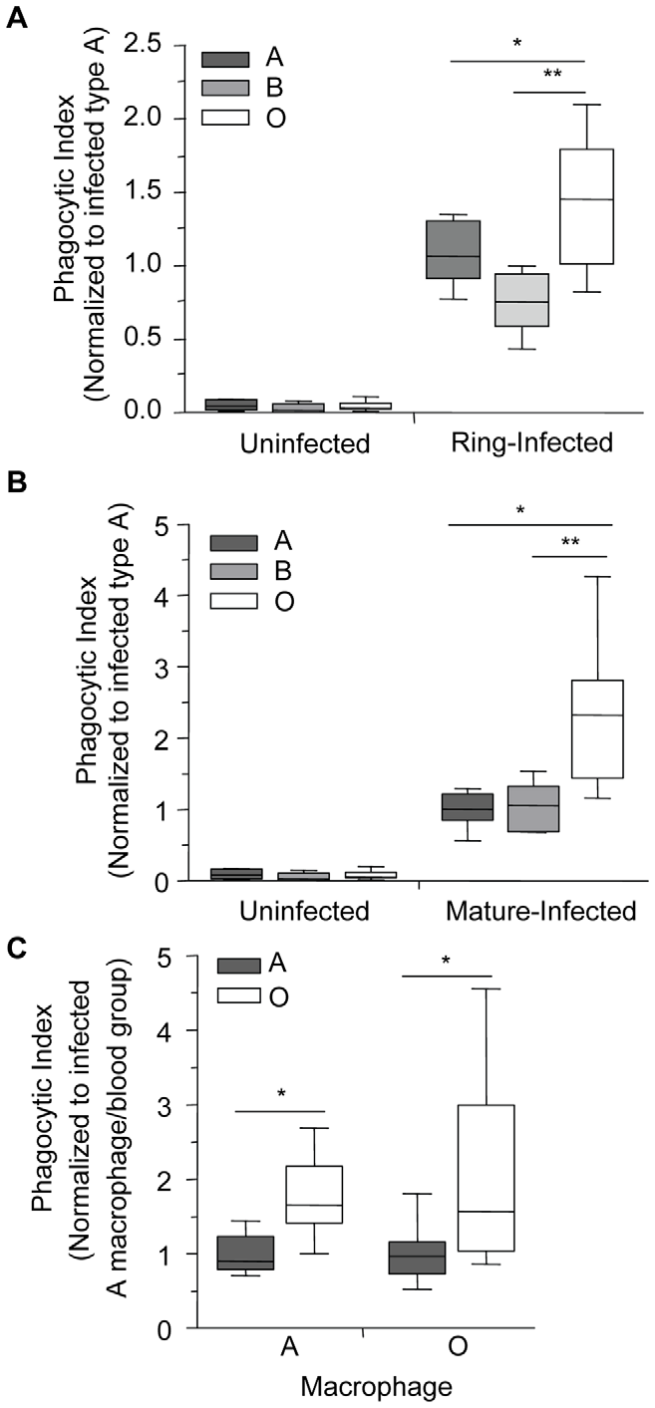
Infected O erythrocytes are phagocytosed more avidly by human macrophages. *P. falciparum* (ITG and 3D7)-infected and uninfected A, B and O erythrocytes were incubated with A and O blood group macrophages for 90 minutes (ring-stage) or for 120 minutes (mature-stage). The phagocytic index was then calculated by determining the number of internalized parasites in ≥250 macrophages and the data were normalized to the average phagocytic index of infected A erythrocytes. (A) Data for ring-stage parasitized erythrocytes are presented as the combined results of two independent experiments. Data for each blood group (n = 3 donors/group) are shown as box-and-whiskers plot, representing interquartile and complete ranges, with the horizontal line in each box indicating the median. There was a significant increase in the phagocytic uptake of ring-stage infected O erythrocytes compared to A and B infected erythrocytes (*p = 0.022, and **p = 0.007, respectively; Student's *t*-test for paired samples with Bonferroni correction for multiple comparisons). (B) Data for mature-stage parasitized erythrocytes represent the combined results of six independent experiments using multiple donors per group (A: n = 6; B: n = 3; O: n = 4) and are shown as box-and-whiskers plots, representing interquartile and complete ranges, with the horizontal line in each box indicating the median. There was significantly enhanced phagocytic uptake of infected O erythrocytes observed compared to A and B infected erythrocytes (*p<0.01 and **p<0.05, respectively; Student's *t*-test with Bonferroni correction for multiple comparisons). (C) Macrophages were isolated from donors of blood groups A and O and phagocytosis assays were run in parallel. The blood group of the infected erythrocytes was found to influence phagocytic uptake, with infected O erythrocytes being preferentially phagocytosed (*p<0.01, two-way ANOVA), whereas the blood group of the macrophage had no effect on the uptake of infected A and O erythrocytes (p>0.05). Data represent three independent experiments normalized to the average phagocytic uptake of infected A erythrocytes by macrophages isolated from blood group A donors.

To test the hypothesis that infected O erythrocytes may be preferentially phagocytosed compared to infected A or B erythrocytes, infected A, B and O erythrocytes at ring- or mature-stage were co-incubated with human monocyte-derived macrophages for 2 hours and the phagocytic index was determined blinded to the erythrocyte blood group. These data were then normalized to the average phagocytic index of infected A erythrocytes. The phagocytic uptake of ring-stage infected O erythrocytes (Figure 2A; 1.43±0.16, mean±SEM) was significantly higher than ring-stage infected A (1.09±0.08, p = 0.022) and B erythrocytes (0.75±0.076, p = 0.007). Similarly, the mean uptake of mature-stage infected O erythrocytes (Figure 2B; 2.3±0.29, mean±SEM) was significantly greater than the uptake of infected A (1.0±0.07, p = 0.001) and infected B (1.02±0.16, p = 0.026) erythrocytes. By contrast there were no significant differences observed in the uptake of control uninfected A, B, or O erythrocytes (Figure 2A, B). Similar results were observed with parasite clone 3D7 (not shown) suggesting that these were not a *P. falciparum* clone-specific phenomenon. We next examined the influence of different blood group macrophage donors on infected erythrocyte uptake at mature-stage. No significant differences were observed in the preferential uptake of infected O erythrocytes by macrophage donors of A versus O blood group (Figure 2C, p = 0.568, two-way ANOVA, main effect: macrophage donor). Macrophages obtained from either group O or A donors displayed enhanced phagocytosis of *P. falciparum*-infected O erythrocytes compared to infected A erythrocytes (Figure 2C, p = 0.002, two-way ANOVA, main effect: blood group). These data indicate that infected O erythrocytes are preferentially cleared by macrophages independent of the macrophage donor blood group.

### Increased phagocytosis of infected group O erythrocytes is observed *in vivo*

**Figure 3:**
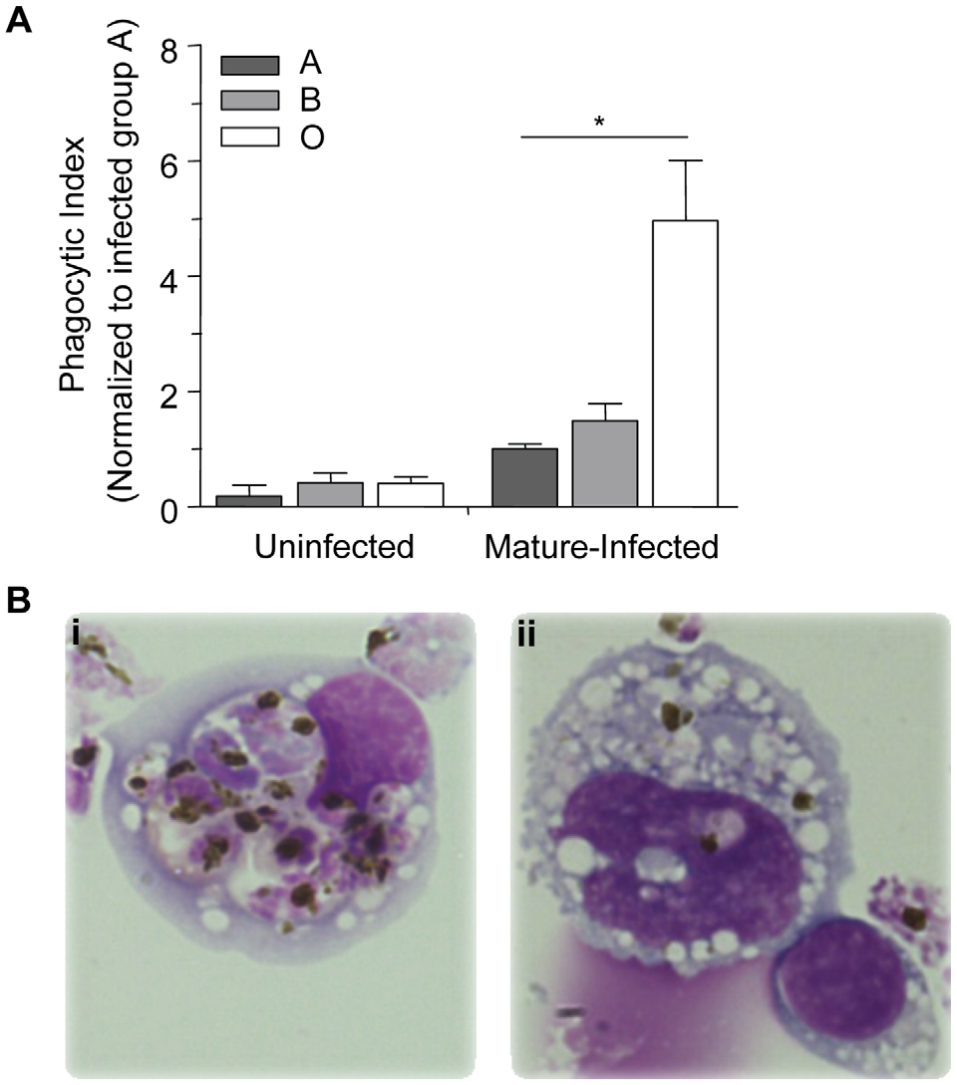
Murine monocytes phagocytose infected O erythrocytes more efficiently *in vivo* than infected A or B erythrocytes. C57BL/6 mice were injected intraperitoneally with 50×10^6^ purified mature-stage *P. falciparum* parasites cultivated in human A, B or O erythrocytes. Three hours after injection, resident monocytes were collected, washed, and plated on glass coverslips. (A) The phagocytic index was calculated by counting the number of internalized parasites within 250 monocytes and the data were normalized to the average phagocytic index of infected A erythrocytes. Data represent three independent experiments using the *P. falciparum* strain ITG and each blood group is represented by at least 3 different donors, A (n = 5), B (n = 3) and O (n = 5). Bar graphs represent the mean±SEM. There was a significant increase in the phagocytic uptake of infected O erythrocytes compared to the phagocytic uptake of infected A or B erythrocytes (*p<0.05; Student's *t*-test with Bonferroni correction for multiple comparisons). (B) Photomicrographs show representative examples of increased uptake of mature-stage infected (i) O erythrocytes, versus mature-stage infected (ii) B erythrocytes. Images were acquired with an Olympus BX41 microscope and an Infinity capture camera at 1000× magnification.

Previous studies have established that murine macrophages express CD36 and bind and mediate the update of *P. falciparum* infected erythrocytes. This model has proven to be useful to investigate the molecular basis for the interaction of CD36 with malaria-infected erythrocytes as well as the interactions of other pattern recognition receptors such as TLRs with their respective cognate ligands *in vitro* [29], [30] and *in vivo* [31]. We used a previously established murine model system [31] to investigate phagocytosis of *P. falciparum-*infected A, B and O erythrocytes *in vivo* by macrophages in the peritoneal cavity of C57BL/6 mice. Three hours after intraperitoneal injection with infected or uninfected (as controls) A, B and O erythrocytes, peritoneal lavage was performed, and phagocytosis of infected and uninfected erythrocytes by peritoneal macrophages was quantified. In agreement with our *in vitro* observations, the phagocytic uptake of infected O erythrocytes (4.97±1.04, mean±SEM) *in vivo* was 3- to 4-fold greater than the phagocytic uptake of infected A (1.00±0.08, p = 0.01) or infected B (1.49±0.29, p = 0.04) erythrocytes (Figure 3A, B). There were no significant differences observed in the uptake of uninfected A, B and O erythrocytes (Figure 3A).

### Macrophage phagocytic index is inversely correlated with A antigen expression on infected erythrocytes

**Figure 4:**
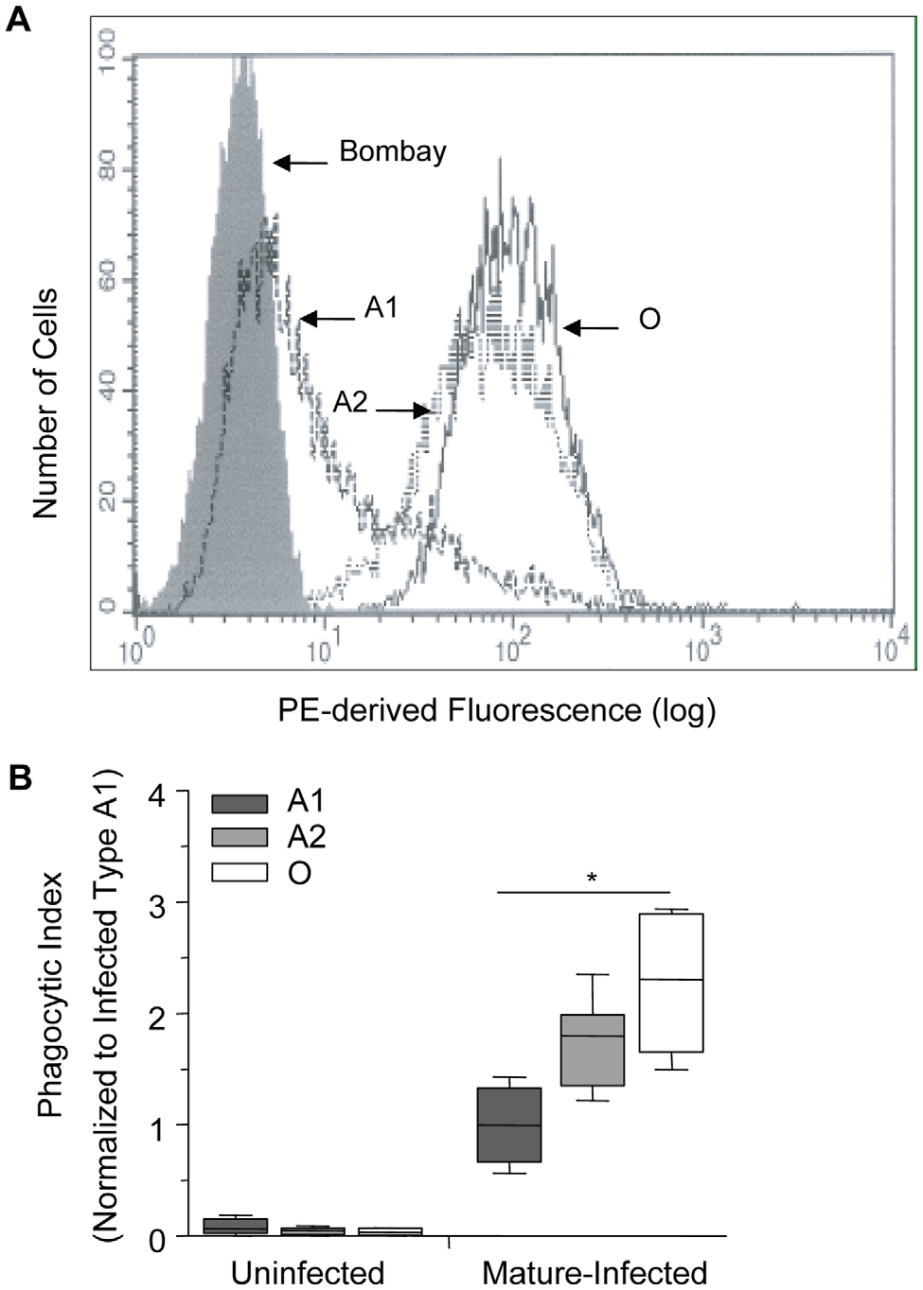
Increasing phagocytosis of erythrocytes correlates with decreasing A antigen. A and O erythrocytes were typed using standard hemagglutinin techniques. Blood group A was further classified into A_1_ or A_2_ subgroups using *Dolichos biflorus* lectin. (A) Flow cytometric testing of A_1_, A_2_, O, and Bombay erythrocytes with anti-H (clone BRIC231) FITC conjugated antibody. FITC-derived fluorescence is displayed on the x axis on a logarithmic scale and the number of cells on the y axis. O (solid black line), A_2_ (dotted black line), A_1_ (dashed black line) and Bombay (filled grey). (B) A_1_, A_2_ and O erythrocytes infected with *P. falciparum* (ITG clone) were incubated with human monocyte-derived macrophages. The phagocytic index was calculated by determining the number of internalized parasitized erythrocytes within 250 macrophages. Data were normalized to the average phagocytic index of infected A_1_ cells. Data represent 3 independent experiments and each blood group is represented by multiple donors, A_1_ (n = 8/group), A_2_ (n = 3/group), and O (n = 8/group). The box plots represent the median, interquartile and complete range. The phagocytosis of infected erythrocytes increased in an H antigen dose-dependent manner (r = 0.80, *p<0.0001; Spearman's correlation test).

There are various subgroups of each ABO blood group, with the two most common subgroups of A being A_1_ and A_2_. The differences between them are both qualitative and quantitative: A antigens are more complex on erythrocytes of the A_1_ phenotype and have an approximate 5× greater antigen site density than on A_2_ cells, thus leaving the latter with a greater number of unmodified H antigens [32], [33]. The quantitative spectrum of H antigen expression is therefore O>A_2_>A_1_>Bombay (where the latter is a genetically H-deficient phenotype) (Figure 4A) [8], [9], [34]. Based on our above observations, we postulated that an increase in H antigen expression, and a corresponding decrease in A antigen levels, would be associated with increased macrophage phagocytosis of infected erythrocytes. Within the spectrum of H antigen expression, we also predicted that phagocytosis of infected A_2_ erythrocytes would be greater than that of infected A_1_ erythrocytes, but less than infected O erythrocytes. In agreement with this hypothesis, we observed a dose-dependent effect with presumed H antigen expression on the phagocytosis of *P. falciparum* infected cells, with phenotypically less A antigen expression (or increasing H antigen expression) associated with increased phagocytosis (Figure 4B; Spearman's correlation, r = 0.80, p<0.0001).

### Enzymatic removal of blood group B antigen increases phagocytosis of infected B erythrocytes

**Figure 5:**
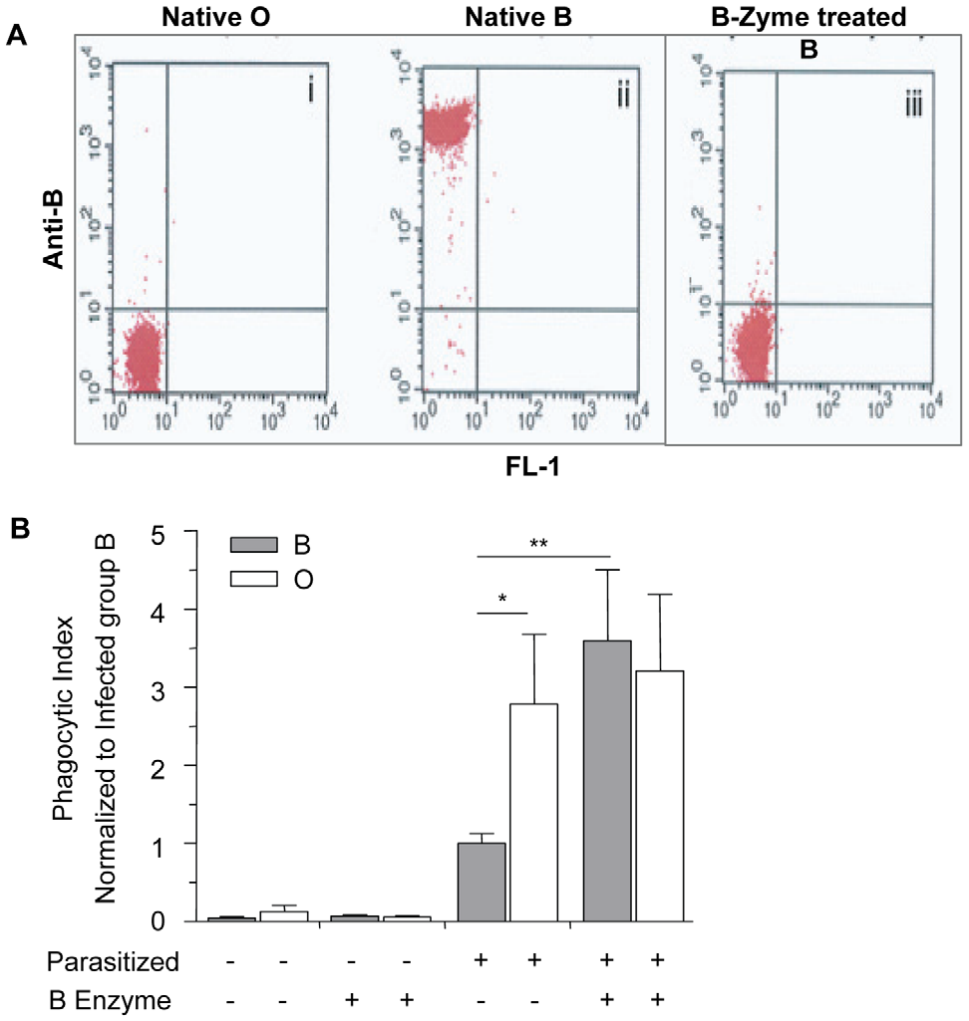
Blood group O (H antigen) status affects the phagocytic uptake of infected erythrocytes by human macrophages. Erythrocytes of blood group O and B were treated with B-zyme, thereby removing the terminal α-1,3-galactose from blood group B antigens. (A) Flow cytometric analysis with anti-B (clone 9621A8) and phycoerythrin (PE)-conjugated rat-anti-mouse kappa as secondary antibody. In the dot plot, the x and y axes represent FL1-derived fluorescence and PE-derived fluorescence, respectively, on logarithmic scales. Results show cleavage of the terminal galactose from B erythrocytes and enzymatic conversion from group B to erythrocytes which type as group O: (i) Native, untreated O erythrocytes, (ii) Native, untreated B erythrocytes, (iii) B-zyme-treated B erythrocytes. Group O cells mock-treated with B-zyme gave identical results to the group O untreated control (data not shown). (B) The phagocytic uptake was determined by counting the number of internalized infected erythrocytes in 250 individual macrophages and data was normalized to the average phagocytic index of infected untreated B erythrocytes. Data represent two independent experiments using the *P. falciparum* ITG clone. Each blood group is represented by four different donors. Bar graphs represent the mean±SEM. Significance was determined by Mann Whitney test with Bonferroni correction for multiple comparisons. There was an observed increase in the phagocytosis of untreated infected O erythrocytes when compared to untreated infected B erythrocytes (*p<0.05). The phagocytic index of the infected, B-zyme-treated B erythrocytes was significantly increased from phagocytic index of infected untreated B erythrocytes (**p<0.01). However, there was no significant difference in the uptake of treated vs. untreated infected O erythrocytes.

To confirm that increased relative H antigen expression and decreased A/B antigen expression was associated with enhanced phagocytosis of infected O erythrocytes, we enzymatically converted B erythrocytes to type as O erythrocytes using a specific glycosidase, B-zyme (*Bacteroides fragilis*, α-galactosidase). This enzyme has been shown to efficiently convert virtually all B antigen on B erythrocytes to H antigen at neutral pH, without changing the sialic acid content on the cell membrane [35]. By treating B erythrocytes with this α-galactosidase, we cleaved the terminal α1–3-linked galactose residues responsible for blood group B specificity, converting these cells serologically to type as blood group O [35]. As controls, untreated group B erythrocytes were treated with the conversion buffer alone and control O erythrocytes were treated with the conversion buffer plus B-zyme. Efficient enzyme conversion of blood group B to O was achieved as demonstrated by flow cytometric analysis (Figure 5A). Positive reactions with anti-B were also demonstrated serologically with B erythrocytes while negative reactions were obtained with O erythrocytes and B erythrocytes converted with B-zyme.

Consistent with our previous findings, there was increased uptake of infected untreated O erythrocytes when compared to infected untreated B erythrocytes (Figure 5B, p = 0.042, Mann-Whitney with Bonferroni correction for multiple comparisons). Moreover, the phagocytosis of infected B erythrocytes was significantly enhanced following cleavage of the B antigen (p = 0.008, Figure 5B) and comparable to mock-treated and infected O erythrocytes. The phagocytic index of infected O erythrocytes was not significantly altered by B-zyme treatment (p>0.05). Additionally, there was no significant difference observed in the uptake of control uninfected, treated or untreated, B and O erythrocytes (Figure 5B).

### Hemichrome deposition and band 3 aggregation is increased in infected O erythrocytes

**Figure 6:**
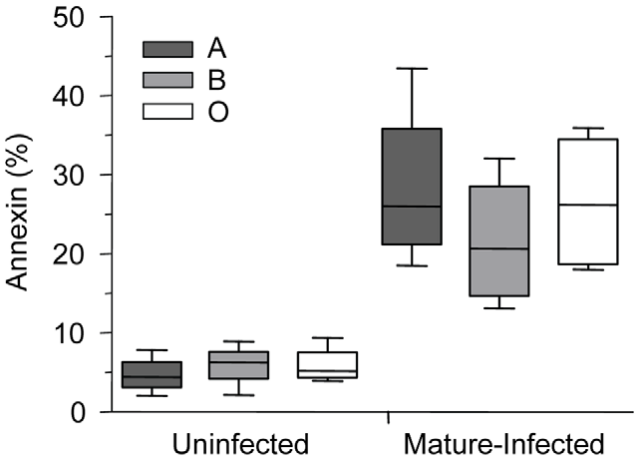
Phosphatidylserine levels on *P. falciparum*-infected A, B and O erythrocytes. Annexin staining of A, B and O uninfected and *P. falciparum*-infected erythrocytes was evaluated. Freshly purified erythrocytes (n = 4 for each group) from healthy donors were left uninfected or were infected with ITG, 3D7 or E8B clones and stained by annexin-V-FITC and propidium iodide and analyzed by flow cytometry. Data are shown as box-and-whiskers plots, representing interquartile and complete ranges, with the horizontal line in each box indicating the median level of percentage of annexin-V positive cells among total cell population. There were no significant differences observed between infected A, B, or O group erythrocytes.

Phagocytic recognition and clearance of senescent erythrocytes has been reported to depend, at least partly, on increased expression of erythrocyte senescence antigens such as phosphatidylserine (PS) and aggregated band 3 [36]. Increased outer leaflet exposure of PS has been associated with enhanced macrophage erythrophagocytosis [36]. To examine potential mechanism(s) underlying enhanced uptake of infected O erythrocytes, we initially investigated *P. falciparum*-induced PS exposure by comparing Annexin-V staining on infected O, A, and B erythrocytes (Figure 6). Although infected erythrocytes had increased PS expression compared to uninfected erythrocytes, there were no significant differences observed in PS levels on infected O compared to infected A and B erythrocytes (Figure 6).

**Figure 7:**
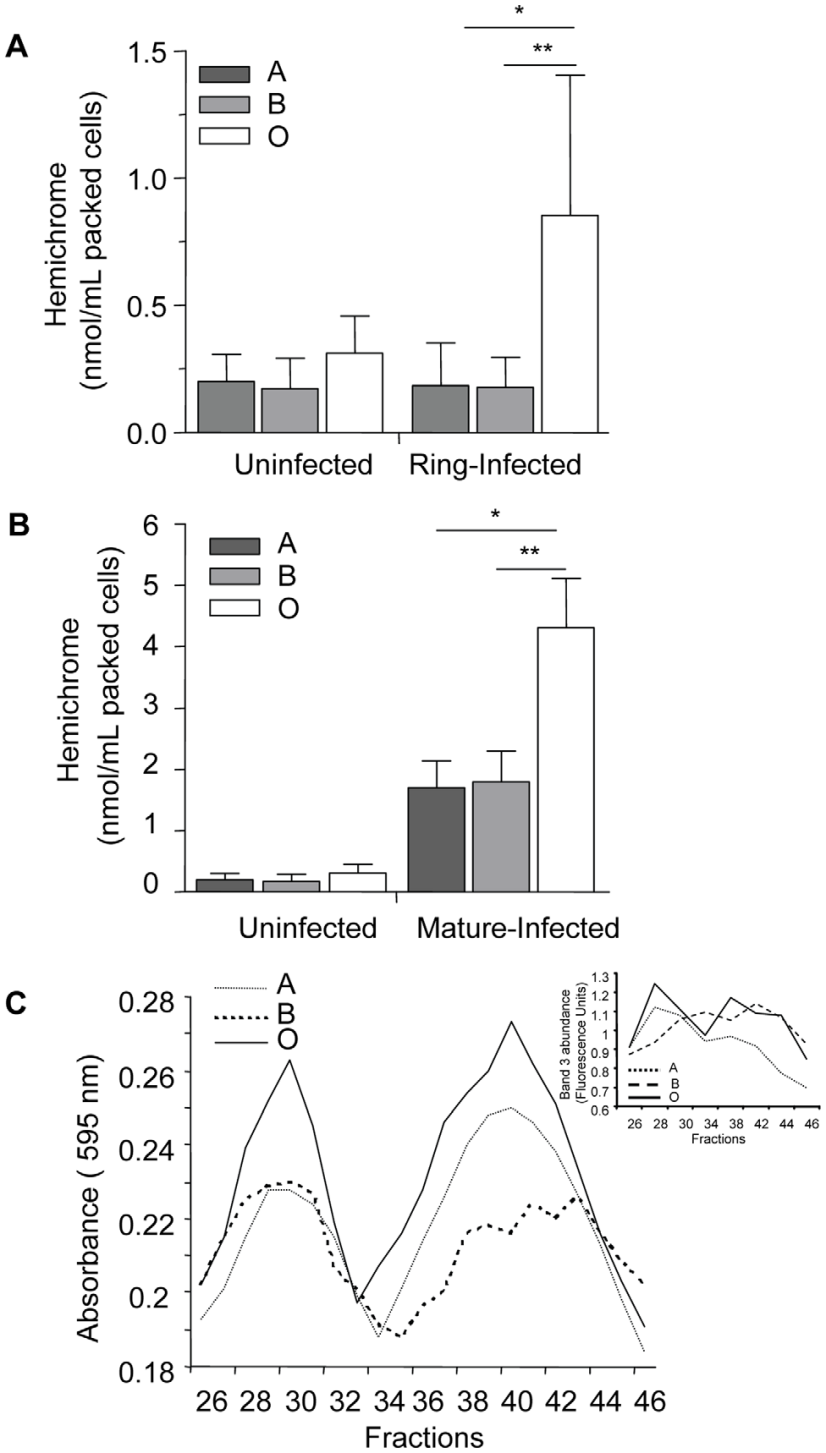
Hemichrome deposition and band 3 aggregation are increased in infected O erythrocytes. Hemichrome levels and band 3 aggregation were measured in uninfected, ring-stage infected and mature-stage infected A, B and O erythrocytes. (A) and (B) Hemichrome data represent the combined results of 6 independent experiments (means±SEM of ≥3 different donors per blood group) using *P falciparum* ITG and 3D7 clones and are expressed in nmol/ml of packed erythrocytes. Compared to infected A and B erythrocytes, hemichrome deposition was significantly increased in ring-stage infected (Figure 7A, *p = 0.005, **p = 0.038, respectively) and mature-stage infected O erythrocytes (Figure 7B, *p = 0.013, **p = 0.024, respectively; Mann Whitney for two-tailed distribution). (C) Gel filtration chromatography effluents of Tween-20 membrane extracts of *P. falciparum*-infected A, B and O erythrocytes (Figure 7C) were analyzed for aggregated band 3 protein as absorbance using the Bradford reagent at 595 nm and eosin-5-maleimide fluorescence indicating the location of band 3 (Figure 7C insert). Increased high molecular weight aggregates of band 3 were observed in infected group O erythrocytes. The results presented are representative of 3 independent experiments.

In contrast, ABO blood groups influenced *P. falciparum*-induced hemichrome deposition and band 3 aggregation. Figures 7A and B shows the presence of membrane-bound hemichromes in uninfected and infected A, B, and O erythrocytes. No differences in hemichrome levels were observed in uninfected erythrocytes maintained in the same culture conditions as infected erythrocytes. However, increased hemichrome deposition was detected in infected ring-stage (p = 0.005 and p = 0.038, Figure 7A) and mature-stage (p = 0.013 and p = 0.024, Figure 7B) O erythrocytes, compared to infected A and B erythrocytes, respectively.

Since hemichromes are thought to bind to and oxidize the band 3 cytoplasmic domain inducing band 3 clustering [25], [36], we compared band 3 aggregation in infected O, A and B erythrocytes using gel filtration chromatography (Figure 7C) and eosin-5-maleimide fluorescence (Figure 7C insert), a specific label for band 3. We observed that extracts from infected O erythrocytes fractionated by gel filtration, display an earlier and higher protein peak in membrane extracts compared to infected A or B erythrocytes. The absorption spectrum of the heme-containing fractions corresponded to that of hemichromes [37]. The same fractions contained aggregated band 3, which was localized by labeling infected ABO erythrocyte membranes with the specific fluorescent band 3 label eosin-5-maleimide as described [38]. The observed chromatographic co-elution of hemichromes and aggregated band 3 is indicative of hemichrome-induced clustering of band 3 [25].

## Discussion

This study provides the first evidence that the phagocytic uptake of *P. falciparum*-infected erythrocytes is influenced by ABO blood group antigens. These data provide a new putative mechanism by which blood group O may contribute to protection against severe malaria. In order to define potential mechanisms of protection associated with blood group O, we investigated *P. falciparum* invasion and growth in ABO erythrocytes, as well as examined a role for differential clearance of infected ABO erythrocytes *in vitro* and *in vivo*. We found no difference in the invasion or maturation of *P. falciparum* parasites in A, B or O erythrocytes. However, we did observe enhanced phagocytosis of infected O erythrocytes by human macrophages (Figure 2) that was attributable to increased hemichrome deposition and band 3 aggregation (Figure 7). This observation was dependent on *P. falciparum* infection as no differences were observed in the baseline uptake of uninfected A, B or O erythrocytes. Preferential phagocytosis of infected O erythrocytes was independent of the donor ABO blood group (Figure 2C). We extended these observations to an *in vivo* model, and demonstrated increased macrophage uptake of infected O erythrocytes *in vivo* compared to infected A or B erythrocytes (Figure 3A, B) [29]. Taken together, our data suggest that there are differences in phagocytic clearance of infected O versus infected A and B erythrocytes which may contribute to reduced parasite burdens and improved malaria outcomes in blood group O individuals.

In order to investigate whether blood group antigens might directly affect phagocytic uptake, we performed phagocytosis assays on infected erythrocytes that varied in their relative expression of H and A antigens. We observed a relationship between phagocytic index and lower levels of immunodominant A or B expression (or higher reciprocal levels of erythrocyte H antigen expression) on infected erythrocytes (Figure 4B). Differences in blood group terminal monosaccharides may influence phagocytosis either directly on the basis of H antigen density, or indirectly by the absence of the A or B antigens. Given that no differences in the baseline uptake of uninfected A, B or O erythrocytes were observed, the preferential phagocytosis of infected O erythrocytes is therefore dependent on *P. falciparum* infection and may be attributable to group-specific differences in parasite-encoded erythrocyte membrane proteins or other *P. falciparum*-induced structural modifications to the erythrocyte membrane. Given the possibility that other inter-individual ABO-associated differences might have accounted for the observed ABO effect on phagocytosis, we examined the uptake of infected erythrocytes that had and had not been enzymatically modified to resemble O erythrocytes (Figure 5). B erythrocytes were chosen since enzymatic conversion of A erythrocytes to O results in not only the common H antigens of types 1 and 2 regularly found on wild type O erythrocytes but also a qualitatively different H antigen of type 3 found on A erythrocytes [39]. In these experiments B- zyme α-glycosidase treatment of B erythrocytes removed the terminal α-1,3-galactose from blood group B antigens, resulting in loss of anti-B recognition and conversion to erythrocytes which type as group O (Figure 5A). Subsequent infection of B-zyme-converted erythrocytes resulted in enhanced macrophage uptake to levels observed with infected wild type O erythrocytes, and different from the levels seen with unmodified B erythrocytes (Figure 5B). B-zyme treatment *per se* was not responsible for this effect, as treatment of uninfected O or B erythrocytes, or infected O erythrocytes, had no affect on their uptake by macrophages.

There are a number of potential explanations for how A/B/H antigens could modify macrophage recognition and uptake. ABO may influence the differential expression of parasite ligands such as PfEMP-1, or the steric accessibility of other parasite-dependent pattern recognition motifs. The H antigen found at high levels on O erythrocytes may alternatively act as a co-receptor to a parasite ligand, or influence other parasite-induced erythrocyte modifications (for example, increased senescence antigen expression by infected O erythrocytes). Recent evidence suggests that ABH antigens can stabilize sialylated glycan clusters on the erythrocyte membrane in a manner that is unique for each blood group [40]. In this way ABH antigens can differentially modulate cellular interactions without being a direct ligand themselves by modifying other cell surface glycans and making them more or less accessible for binding. Cohen et al. have shown that by stabilizing such structures, ABH antigens can also modulate interaction with pathogens such as *P. falciparum* [40]. Therefore, it is possible that ABH antigens may non-covalently alter the expression or presentation of other cell surface glycans including parasite encoded proteins such as *P. falciparum* erythrocyte membrane protein-1 (PfEMP-1). PfEMP-1, an important parasite virulence factor [41], has also been shown to demonstrate differential expression on the erythrocyte membrane in erythrocyte disorders, including hemoglobin C, associated with protection to severe malaria [42]. It is therefore plausible that modified expression of PfEMP-1 or other parasite ligands on O erythrocytes, results in increased interaction with macrophage pattern recognition and phagocytic receptors and enhances uptake. Our data are consistent with a model whereby infected O erythrocytes bind more avidly to phagocytic cell receptors resulting in enhanced uptake (Figure S1).

In addition to a putative role for ABH antigens in modifying parasite-erythrocyte interactions, phagocytic recognition and clearance of erythrocytes have also been associated with increased expression of erythrocyte senescence antigens such as aggregated band 3 and phosphatidylserine (PS) [43], [44]. Although PS exposure has been reported to be elevated in variant erythrocytes, (e.g., sickle cell trait) where it may serve as a senescence signal for accelerated clearance [45], we found no significant difference in *P. falciparum*-induced PS expression on infected red cells to account for the observed preferential uptake of infected O erythrocytes.

With respect to band 3, there are approximately 1 million ABH glycan antigen sites on each erythrocyte, and many are presented on this protein [43]. Increased band 3 aggregation has been reported in sickle cell and β-thalassemic erythrocytes, contributing to erythrocyte membrane modification and enhanced phagocytic uptake by macrophages. Whether the absence of immunodominant sugars is more permissive to malaria-induced band 3 aggregation is unknown, as ABO effects have not previously been specified in such studies. In the present study we observed increased hemichrome formation and band 3 aggregation in infected O erythrocytes compared to infected A and B erythrocytes. The mechanism by which O erythrocytes might be more susceptible to malaria-induced oxidant stress is not known. Erythrocytes under increased oxidative stress, such as that induced by malaria parasite invasion and growth, may show preferential oligomerization/phosphorylation of less-glycosylated band 3 fractions [46]. This possibility is consistent with reports that band 3 displays an increased tendency to cluster in congenital dyserythropoietic anemia type 2 which is characterized by band 3 under-glycosylation, and with the irreversible cross linking observed in poorly glycosylated band 3 fractions in G6PD-deficient erythrocytes [46]–[48]. Glycosylation of band 3 appears to be a restraint to its oxidative cross-linking, clustering and subsequent phagocytic uptake. Collectively these observations provide a putative molecular mechanism for the observed enhanced uptake of infected O erythrocytes.

In summary, we have demonstrated a novel mechanism by which blood group O may contribute to protection against severe disease. The present model is complementary to, and not incompatible or inconsistent with, the decreased rosetting of infected O erythrocytes reported by others [12]. Both increased phagocytosis and decreased rosetting of blood group O may contribute functionally to reduced parasite burden, decreased infected erythrocyte adhesion to the endothelium and decreased microvascular obstruction, all of which are believed to play important mechanistic roles in the pathophysiology of severe falciparum malaria.

## Materials and Methods

### Ethics statement

Whole blood was donated from healthy malaria-naïve individuals after informed consent using a protocol approved by the University Health Network Research Ethics Board. Animal use protocols were reviewed and approved by the Faculty of Medicine Advisory Committee on Animal Services at the University of Toronto according to the Guide to the Care and Use of Experimental Animals (Canadian Council on Animal Care, 1993).

### Reagents

Endotoxin-free RPMI 1640 and gentamicin were purchased from Invitrogen Life Technologies (Burlington, ON, Canada). Human AB serum was purchased from Wisent Inc (St-Bruno, Quebec, Canada). Diff-Quik staining kit and fetal bovine serum (FBS) were purchased from Fisher Scientific (Ottawa, ON, Canada). FBS and human AB serum were heat-inactivated for 30 minutes at 55°C before use. Alanine was purchased from Sigma Aldrich (Oakville, Ontario, Canada). Mycoplasma removal agent was purchased from MP Biochemical (Solon, Ohio, USA). Ficoll-Paque and Percoll were purchased from GE Healthcare (Baie D'Urfé, Québec, Canada). NOVACLONE blood grouping reagent was purchased from Dominion Biologicals Ltd (Dartmouth, Nova Scotia, Canada). All other reagents were purchased from common commercial sources.

### Mice

C57BL/6 mice used in this study were 6–10 weeks old and were purchased from Charles River Laboratories (Hollister, CA) and maintained under pathogen-free conditions with a 12-h light cycle.

### *P. falciparum* culture

*P. falciparum* (clones ITG, 3D7 and E8B) was cultured as previously described [47]. Cultures were treated with *Mycoplasma*-Removal Agent, confirmed to be *Mycoplasma*-free (MycoAlert *Mycoplasma* Detection Kit, Lonza) and synchronized by alanine treatment.

### Erythrocyte and serum isolation

Whole blood was obtained from hematologically healthy laboratory staff members (11 group A, 4 group B and 6 group O). Individuals with underlying red cell traits or disorders, or previous malaria exposure were excluded. Erythrocytes were separated from whole blood as previously described [27]. Briefly, whole blood was layered on an 80% Percoll gradient [80% (vol/vol) Percoll, 6% (w/v) mannitol, 10 mM glucose and 10% (vol/vol) PBS 10×] and spun for 15 minutes at 3000 RPM at 24°C. The erythrocyte pellet was collected and washed in R-0G media (RPMI 1640 medium supplemented with 10 mM glucose and 10 g/L gentamicin) and resuspended in equal volumes of parasite growth medium R-10G (RPMI-1640 containing 20 mM glucose, 2 mM glutamine, 6 g/L HEPES, 2 g NaHCO_3_, 10 g/L gentamicin, 10% human AB serum and 1.35 mg/L hypoxanthine, pH 7.3). Serum was isolated by centrifugation at 1500 RPM for 10 minutes at 24°C, and 200 µl aliquots were stored at −20°C for future use. Each aliquot was thawed only once and discarded after use.

### ABO blood group typing

Blood samples were tested by standard hemagglutination techniques with commercially available anti-A and anti-B reagents approved for diagnostic use [39]. Donors expressing A antigens were further typed using *Dolichos biflorus* lectin to differentiate between the A_1_ and A_2_ subgroups.

### Macrophage isolation

Human monocytes were purified from the peripheral blood of healthy donors and cultured on glass cover slips in 24-well polystyrene plates as previously described [49]. Briefly, whole blood was diluted 1∶1 with warm PBS, layered onto Ficoll (25 mL/15 mL) and centrifuged at 1800 RPM for 30 minutes at 20°C. The peripheral blood mononuclear cell (PBMC) layer was washed 3 times with cold RPMI-1640 and resuspended in R-10G FBS media (RPMI-1640 medium containing L-glutamine and HEPES supplemented with 10% heat-inactivated FBS and 25 mg/L gentamicin). 1.25×10^6^ PBMCs were pipetted onto 24-well plate containing coverslip and incubated at 37°C for 1 hour. Wells were washed twice to remove non-adherent cells and the monocytes were cultured in R-10G FBS for 5 days at 37°C to allow differentiation into macrophages.

### Parasite invasion and maturation in ABO erythrocytes

To assess parasite invasion and maturation, schizont stage *P. falciparum*-infected erythrocytes from synchronized cultures were purified on a Percoll-mannitol gradient [27], [50] and mixed with erythrocytes of different blood groups (A, B and O) in R-10G as described [27]. Invasion of erythrocytes was assessed at 24 hours, and 72 hours, and maturation was assessed at 48 hours, and 96 hours. Slides were stained with Diff-Quik, and 1000 erythrocytes were examined microscopically. Percent parasitemia was determined as follows: *[number of parasites÷number of total erythrocytes counted]×100*.

### Phagocytosis assay with human macrophages

Uninfected and infected ring-stage or mature-stage parasitized erythrocytes were incubated with 50% fresh autologous serum for 30 minutes at 37°C. Erythrocytes were then washed twice, resuspended at 10% hematocrit, and incubated with macrophages adherent to glass coverslips at a target-to-effector ratio of 40∶1(ring-stage) or 1∶20 (mature-stage). Phagocytosis assays were performed as described previously [27] and were counted and analyzed blinded to the erythrocyte blood group.

### Murine model of phagocytosis

To assess phagocytosis of infected A, B and O erythrocytes *in vivo*, 50×10^6^ infected erythrocytes, or uninfected erythrocytes as a control, were injected into the peritoneal cavity of C57BL/6 mice as previously described [29]. Three hours after injection, peritoneal cells were collected, and washed with R-0G media. The cells were then suspended in cold water to lyse and remove non-internalized erythrocytes. Cells were then resuspended in 500 µl of R-10G media. 150 µl aliquots of peritoneal cells from each mouse were placed on a glass coverslip in a 24 well plate, allowed to adhere for 30 minutes at 37°C and stained with Diff-quick. In addition, 200 µl of the suspension were cytospun at 800 RPM for 10 minutes and stained with Diff-quick. Images from these slides were acquired with an Olympus BX41 microscope and an Infinity2 camera at 1000× magnification.

### Analysis of H antigen status

Flow cytometric detection of H antigens was performed as previously described [34], using a FITC-conjugated monoclonal anti-H (BRIC231) antibody. Blood groups A_1_, A_2_, O and H negative control (Bombay), were tested simultaneously and ten thousand events were collected. In the histogram FITC-derived fluorescence is displayed on the x axis on a logarithmic scale and the number of cells is on the y axis.

### Enzymatic conversion of B erythrocytes and post-treatment analysis of B antigen status

Removal of B antigens was achieved as previously described [34]. Briefly, erythrocytes were prewashed 1∶1 and 1∶4 vol/vol in glycine buffer (200 mM glycine and 3 mM NaCl, pH 6.8). The conversion reaction consisting of a 30% suspension of erythrocytes in glycine buffer with addition of bacterially-derived GH110 family α-galactosidase (B-zyme from *Bacteroides fragilis*) [34], [51]. The bacterial glycosidase was incubated for 60 min during gentle mixing at 26°C, followed by 4 repeated washing cycles with 1∶4 vol/vol of saline by centrifugation at 1000 RPM. To verify the removal of B antigen after enzyme conversion flow cytometric detection of ABO antigens was performed as recently outlined [51], using the IgM anti-B clone 9621A8 (Diagast, Loos, France) as primary antibody and phycoerythrin (PE)-conjugated rat-anti-mouse Ig kappa light chain (Becton Dickinson, San Jose, CA, USA) as secondary antibody. Treated and untreated erythrocytes were tested simultaneously and control cells of known phenotype (B, B_weak_ subgroup and O cells were included in each run to confirm sensitivity and specificity of the assay, as previously shown) [51]. Ten thousand events were collected and log fluorescence data was gated on a linear forward scatter versus linear side scatter dot plot.

### Extraction of ABO erythrocyte membranes with nonionic detergent, quantification of hemichromes, assay of aggregated band 3 and gel-fitration chromatography

A, B and O uninfected and *P. falciparum* ring-stage and mature-stage infected erythrocytes were washed in PBS supplemented with 0.5% BSA and 0.1% azide and lysed with Tris-HCl/EDTA (pH 8.0) in the presence of protease inhibitors cocktail (Roche Diagnostics GmbH). Where indicated, erythrocytes were incubated for 30 minutes at room temperature in the dark in PBS-glucose containing 10 µmol/L eosin-5-maleimide in order to label band 3 in situ [52]. Membrane pellets were extracted as described [38]. Hemichromes were quantified using the Winterbourne equation [37]. Tween-20 detergent-extracted membrane proteins (500 µL) were then loaded onto Sepharose CL-6B column equilibrated with 10 mmol/L Tris buffer and separated at a flow rate of 0.760 mL/min. The effluent was collected in 1.2 mL fractions. Total proteins in the fractions were assayed using Bedford reagent at 595 nm and labeled band 3 was assayed using fluorometry (Ex-522 nm and Em-550 nm). Aggregated band 3 was assayed in the Tween-20 fractions of uninfected and mature stage-separated infected erythrocytes previously labeled by the band 3-specific fluorescent label eosin-5-maleimide as described [53]. In order to quantify the percentage of aggregated band 3, eosin-5-maleimide-labeled band 3 in Tween-20 fractions, the fluorescence value measured in the high-molecular-weight fractions was normalized to the total fluorescence measured in all fractions.

### Statistical analysis

Statistical analysis was performed using GraphPad Prism 4 software (San Diego, CA, USA). To confirm the normal distribution of data, all continuous data sets were assessed using the Kolmogorov-Smirnov test. Data sets that displayed normal distribution were analyzed by Student's *t*-test (two-tailed) or a one-way ANOVA as appropriate. Data sets that did not display normal distribution were analyzed by the Mann-Whitney rank sum test. Multiple comparisons were corrected using the Bonferroni method. A general linear model was used to analyze experiments with multiple independent variables (e.g., macrophage and erythrocyte group). To test the dose-dependent effect of the A and H antigen, we used a Spearman's rank correlation on the individual data points (phagocytic index) correlate with decreasing level of A antigen. Data are presented as box plots representing the median, inter-quartile range and range or as bar graphs representing the mean±SEM.

## Supporting Information

Figure S1 **Cytoadherence of ItG- and 3D7-parasitized erythrocytes.** ItG- and 3D7-infected O erythrocytes have increased adhesion compare to non-O (A and B) infected erythrocytes. Parasitized non-O erythrocytes adhered less well (∼28% reduction in adherence) to macrophages than parasitized O erythrocytes. Adherence of mature-infected O erythrocytes was significantly higher than mature infected non-O erythrocytes (*p = 0.039, n = 3 experiments, 3 donors for each blood group). Non-infected erythrocytes showed no difference. Cytoadherence assays were performed using human macrophages as described in Text S1. Cytoadherence is shown as the number of infected erythrocytes bound per macrophage. Infected and non-infected erythrocytes were opsonized with autologous serum. Data represent 3 independent experiments normalized to the mean number of infected A erythrocytes bound by macrophages. The box plots represent the median, interquartile and complete range. Significance was determined by Mann-Whitney for two-tail distribution.
(TIF)

Text S1 **Cytoadherence assay.**
(DOC)
