# Supplementary figures and images for: ABO Blood Groups Influence Macrophage-mediated Phagocytosis of Plasmodium falciparum-infected Erythrocytes
Source: PLoS Pathog. 2012 Oct 11;8(10):e1002942. doi: 10.1371/journal.ppat.1002942 (PMC3469569; doi:10.1371/journal.ppat.1002942)

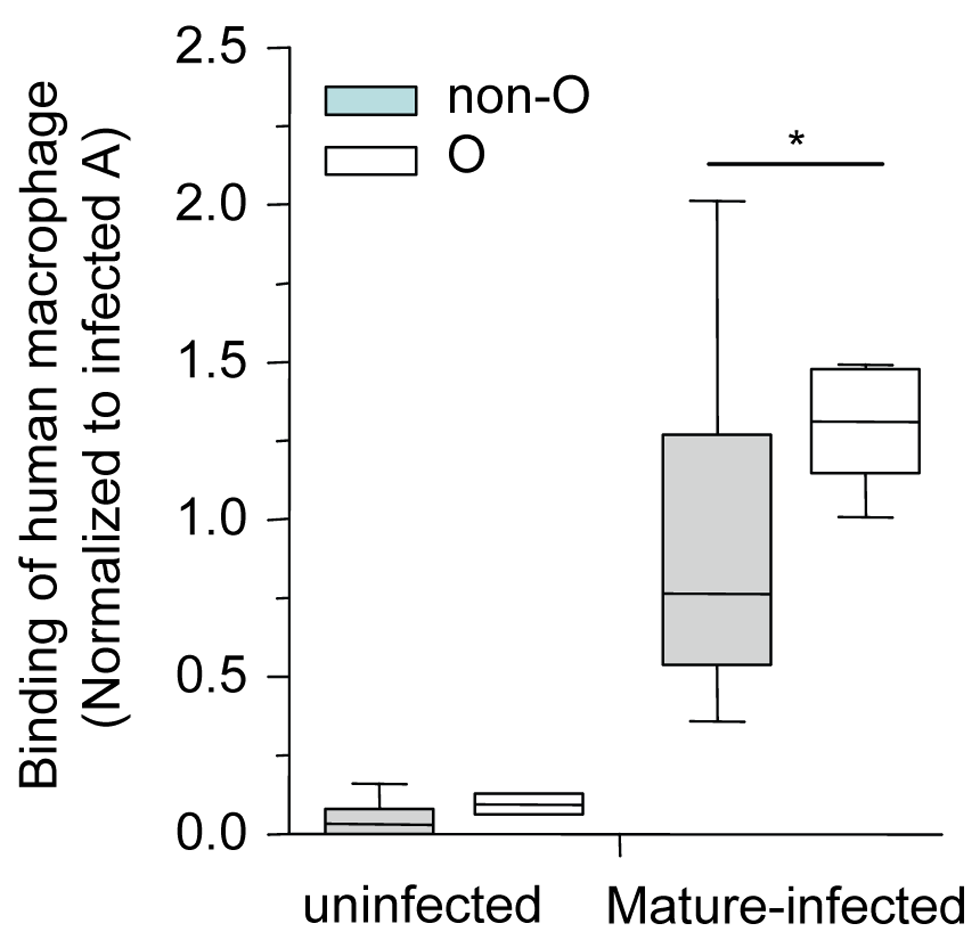

Supplement: Figure S1 — Cytoadherence of ItG- and 3D7-parasitized erythrocytes. ItG- and 3D7-infected O erythrocytes have increased adhesion compare to non-O (A and B) infected erythrocytes. Parasitized non-O erythrocytes adhered less well (∼28% reduction in adherence) to macrophages than parasitized O erythrocytes. Adherence of mature-infected O erythrocytes was significantly higher than mature infected non-O erythrocytes (*p = 0.039, n = 3 experiments, 3 donors for each blood group). Non-infected erythrocytes showed no difference. Cytoadherence assays were performed using human macrophages as described in Text S1. Cytoadherence is shown as the number of infected erythrocytes bound per macrophage. Infected and non-infected erythrocytes were opsonized with autologous serum. Data represent 3 independent experiments normalized to the mean number of infected A erythrocytes bound by macrophages. The box plots represent the median, interquartile and complete range. Significance was determined by Mann-Whitney for two-tail distribution. (TIF) [file ppat.1002942.s001.tif]
