# Supplementary material for: ABO Blood Groups Influence Macrophage-mediated Phagocytosis of Plasmodium falciparum-infected Erythrocytes
Source: PLoS Pathog. 2012 Oct 11;8(10):e1002942. doi: 10.1371/journal.ppat.1002942 (PMC3469569; doi:10.1371/journal.ppat.1002942)
Supplement: Text S1 — Cytoadherence assay. (DOC) [file ppat.1002942.s002.doc]

**Supporting information**

**Text S1. Cytoadherence assay.** Human monocytes were purified from the peripheral blood of healthy donors and cultured on glass cover slips in 24-well polystyrene plates as previously described [49]. After 5 days at 37ºC to allow differentiation into macrophages, synchronized A, B and O erythrocytes infected by mature-stage parasites were added at a ratio of 20 parasitized erythrocytes to 1 macrophage in 0.5mL of R-0G and incubated with rotation for 120 min at 4°C. Unbound infected erythrocytes were removed by washing and erythrocytes-macrophages are stained by Diff-Quik. Cytoadherence was quantified microscopically by counting the number of bound erythrocytes per macrophage.
